# Optimization of a recombinant BlaR-CTD protein formulation using the response surface methodology

**DOI:** 10.1186/s13036-023-00399-9

**Published:** 2024-01-11

**Authors:** Mohadeseh Haji Abdolvahab, Mojdeh Safari, Farkhonde Hasannejad, Nika Asefi, Alireza Salimi, Mahboobeh Nazari

**Affiliations:** 1 Recombinant Proteins Department, Breast Cancer Research Center, Motamed Cancer Institute, ACECR, Tehran, Iran; 2 Department of Medical Nanotechnology, School of Advanced Technologies in Medicine, Tehran University of Medical Sciences, Tehran, Iran; 3 Genetic Department, Breast Cancer Research Center, Motamed Cancer Institute, ACECR, Tehran, Iran; 4 Department of Advanced Technologies, School of Medicine, North Khorasan University of Medical Science, Bojnurd, Iran; 5 Endocrine Research Center, Institute of Endocrinology and Metabolism, Iran University of Medical Sciences, Tehran, Iran; 6 Nanobiotechnology Research Center, Avicenna Research Institute, ACECR, Tehran, Iran

**Keywords:** β-Lactam antibiotics, BlaR-CTD, Stability, RSM, CCD, Design of Experiments

## Abstract

The sequence of a carboxy-terminal of the β-lactam sensor-transducer protein (BlaR-CTD) from Bacillus licheniformis ATCC14580 was extracted from US7745193B2 patent and expressed in E. coli using pColdI vector as a soluble His-tag recombinant protein. In this study, several excipients were used to improve the stability of recombinant BlaR-CTD and obtain the optimal formulation for this protein using response surface methodology (RSM)/ Central Composite Design (CCD). Total protein concentration was measured by UV spectroscopy and the Bradford test. A total of 7 various factors were designed using four different excipients including Glycerol, Sucrose, Triton x-100, and Tween-20, and three different buffers like Tris, Borate, and PBS. By obtaining suitable excipients and buffer i.e. glycerol and sucrose, pH ranging from 7 to 9 were evaluated. The pH 7.62, glycerol 15.35%, and sucrose 152.52 mM were determined as the most suitable for improving the thermal stability of recombinant BlaR-CTD.

## Introduction

Penicillin binding proteins (PBPs) are a group of receptors with the affinity to bind penicillin, a usual component of bacterial cell wall. Most of the β-lactam antibiotics can bind to PBPs. The beta-lactam sensor-transducer (BlaR) is an integral PBP membrane protein which transduces the signal to induce β-lactamase synthesis leads to the degradation of the β-lactam antibiotics [1]. The C-terminal domain of BlaR (BlaR-CTD) situated in the extracellular region of the membrane can bind to the β-lactam antibiotics with its serine active site [2–5]. The crystal structure of *B. licheniformis* BlaR-CTD has been experimentally solved [6]. Although the signaling mechanism of BlaR-CTD is not fully understood, it has been confirmed that BlaR-CTD is acylated by the β-lactam antibiotic targeting the cell. In several screening assays, nano-gold particles labeled with BlaR-CTD protein have been used to recognize the β-lactam residues of biological liquid [7]. The BlaR-CTD of *B. licheniformis* can be expressed in *E. coli* as a recombinant protein [4, 8]. Escherichia coli has been widely used in recombinant DNA technology to produce recombinant proteins in recent decades. The main factors behind the popularity of using *E. coli* are adequate genetic information, rapid growth in a short time, and low nutritional demand. In addition, different *E. coli* strains have been introduced to express foreign genes via simple procedures. Compared to the eukaryotic expression system, *E. coli* can express our desired protein at a 2–threefold lower cost, an essential industrial benefit.

Recombinant proteins are essential in various applications, ranging from therapeutics to high-quality chemical compounds. Therefore, the distinct structures of recombinant proteins heavily influence their effectiveness and safety [9]. One of the most crucial factors that should be considered for optimal conditions is preserving ingredients influencing recombinant proteins under biological processes. Thus, the desirable structure of vaccine therapeutics provides opportunities to produce new vaccines. In the Falahati HIV-1 vaccine study, different conditions, such as changes in temperature, pH, and denaturation, profoundly affected the flexibility of recombinant proteins. Therefore, they reported that recombinant proteins in the presence of additive biocompatible compounds, such as zinc, can conserve their stability [10]. As recombinant proteins mostly are potent to be aggregated following by losing activity, find a suitable formulation is significant to maintain their stability and functionality in the biotech industry. Optimal conditions of biological procedures can be defined by statistical methods through experimental plans. One of the statistical experimental plans widely used is central composite design (CCD). This methodology offers an advanced platform to identify several variables with positive effect on the biological process. In this experimental design, a group of individual parameters are studied over a specific range of doses to determine the optimum levels, the effect of separate variables, and the relation among parameters [11]. To increase the stability of purified recombinant BlaR-CTD protein, Design of Experiments (DOE) via CCD was applied. DOE is a statistical tool that examines factors and their different levels simultaneously by a reduced number of experiments via fractional factorial models such as response surface methodology (RSM) to evaluate more relevant interactions among variables [12]. RSM has been used in various studies to optimize the formulation and expression of recombinant proteins, including soy protein-based edible film, antibody fragments, interferon-beta, and surface immunogenic protein of Group B Streptococcus. RSM helps identify the critical factors that influence protein expression and stability, such as post-induction temperature, IPTG concentration, and cell growth conditions [13–16]. In a study carried out by Aghaeepoor et al., RSM was applied successfully to create optimal conditions for the production of recombinant Streptokinase [17]. In another study, Díaz-Dinamarca investigated the optimized expression condition of recombinant surface immunogenic protein of group B Streptococcus by response surface method to improve humoral immunity. According to the results, RSM employed to optimize the expression of recombinant proteins with immunogenic capacity. They stated that RSM is a suitable approach in the assessment of vaccines in preclinical phase [16]. Therefore, RSM seems to be a suitable method to optimize the formulation of recombinant BLaR-CTD protein.

Measuring protein aggregation and stability is crucial for assessing the quality of protein formulations. Protein aggregates are often irreversible and stable, posing challenges to the development of effective strategies to probe and quantify protein aggregation, including stability in vitro. High-throughput biophysical approaches are available for measuring protein aggregation and stability, and for screening the addition of specific ligands, additives, or excipients to reduce protein aggregation and improve stability [18]. These approaches can evaluate conformational and colloidal stability, and predict and mitigate protein aggregation in the early stages of drug development and bioprocessing [19]. Additionally, UV–Vis spectrophotometry can be used to identify protein aggregation resulting from different stress conditions, providing a quick method to characterize protein aggregation and assess protein quality [20].

Potential protein aggregation could be measured by looking at a decrease in the ratio of OD280nm/ OD260nm and an increase in OD at 350 nm [21, 22]. It is essential to monitor the OD280/OD260 ratio and OD at 350 nm throughout the protein purification process to ensure the presence of minimal aggregates and maintain protein quality.

To define the effect of each factor on preserving recombinant BLaR-CTD protein against aggregation during incubation at 37 °C and obtain the most suitable excipients to improve the thermal stability of this recombinant protein, the OD at 350 nm as well as the ratio of OD280nm/ OD260nm need to be measured by UV spectroscopy. Hence, we conducted a comprehensive study to determine the optimum formulation for recombinant BLaR-CTD protein using several excipients. We sought to identify the effects of several factors on the thermal stability of recombinant BlaR-CTD protein based on the DOE for 350 and 280/260 wavelengths using the RMS/ CCD approach.

## Material and methods

### Chemicals, host strains and culture media

Isopropyl β-d-1-thiogalactopyranoside (IPTG) and restriction enzymes were purchased from Fermentas (Waltham, MA, USA). The pCold II DNA vector was obtained from TAKARA BIO (Shiga, Japan). Ni SepHarose® High Performance affinity column was supplied from GE Healthcare (Buckinghamshire, LC, UK). Competent *E. coli* Bl21 (DE3) was purchased from ThermoFisher scientific (Waltham, Massachusetts, United States). Tryptone and yeast extract used as a culture medium were obtained from ibresco lifescience (Sigma-Aldrich, Missouri, United States).

### BlaR-CTD Construct preparation

The sequence of BlaR-CTD (789 bp) was obtained from published patent i.e. US7745193B2, synthesized and amplified by PCR using forward and reverse primers containing NdeI and HindIII restriction sites respectively by Pishgam co. (Iran) (Table 1). The PCR product was double digested with NdeI/HindIII and subcloned to digested/ dephosphorylated pColdII.

**Table 1:** Primer’s sequence used for the amplification of BlaR-CTD

primer	sequence
F-NdeI-BLA primer	TTTAACATATGCAGAAAGAAACCCGCTTCCTGCCA
R-HindIII-BLA primer	TTTAAGCTTAATGATGATGATGATGGTGGCGGGAAA

### Protein expression and purification

*E. coli* BL21 gold (DE3) bacteria was transformed with confirmed construct. The selected clone was cultured in 2 ml TB broth including 12μg/ml tetracycline and 100 μg/ml ampicillin for 14–16 h at 37 °C, 240 rpm. Afterward, 200 ml of TB broth was inoculated with pre-culture media, and incubated at 37 °C for 7–8 h with the shaking speed of 240 rpm until reaching the stationary phase with OD (optical density): 2.5. We quickly decreased the temperature of the culture medium to less than 15 °C by placing the flask in cold water and incubate at 15 °C for 1 h at 240 rpm. Then, the sterile IPTG was added to final concentration of 0.2 mM and incubated overnight with 240 rpm shaking at 15 °C. Bacterial culture centrifuged at 6000 rpm for 20 min. The supernatant is discarded and the precipitate was washed with the distilled water. The bacteria pellet was vortexed and completely dissolved in lysis buffer (NaCl 300 mM, Tris 50 mM and Imidazole 10 mM). Then, the suspended pellet was sonicated with 10 times 30 s sonication pulses 1min rest on ice. After sonication, the bacterial debrides were centrifuge at 9000 rpm for 20 min, and if the supernatant was not clear, the centrifuging was repeated again for 10 min (12000rpm/10min). The soluble fraction of recombinant BlaR-CTD is in the supernatant.

The supernatant was purified using Ni Sepharose High Performance affinity chromatography. BlaR-CTD protein containing 6 × His tag at both the N-terminus and C-terminus enhanced its binding affinity to the nickel-charged resin. At this stage, the supernatant was run into the column using basis buffer (NaCl 300 mM, Tris 50 mM and Imidazole 10 mM), and His-tagged BlaR-CTD proteins stuck to the nickel-charged resin. After 2 times washing steps with wash buffer (NaCl 300 mM, Tris 50 mM and Imidazole 40 mM), the impurities were washed out. By adding elution buffer (NaCl 300 mM, Tris 50 mM and Imidazole 300 mM), recombinant BlaR-CTD proteins were detached from nickel-charged resins and the eluate was collected.

### Protein analysis with SDS-PAGE and western blot

Recombinant BlaR-CTD expression and purification were evaluated by SDS-PAGE method. Analysis of expressed BlaR-CTD was performed by observing the protein band with a size of ~ 30 kD in SDS-PAGE gel. Afterward, western blot analysis was carried out to verify the SDS-PAGE results. The expressed and purified BlaR-CTD before and after formulation was separated on a 12% SDS-PAGE and transported to a nitrocellulose membrane. After shaking for 1h in 5% skimmed milk solution, the nitrocellulose membrane was washed three times in PBS (1X, pH 7.4) and tween 20 (5% V/V), followed by incubation for 1h to a 1/750 dilution of mouse anti-his tag HRP conjugated (Sina Biotech, Iran) in PBS to reach the final concentration of 4 μg/mL. After washing, the membrane was exposure to the ECL western blotting substrate and HRP-dependent signal was recognized by radiography.

### Enzyme-Linked Immunosorbent Assay (ELISA)

The functionality of the purified BlaR-CTD was determined by ELISA. To this aim, 800 µg/ml BSA-Penicillin conjugate in PBS was added to each well. After washing the plate with 250 µl wash-buffer (0.05% (w/v) tween-20 in 1 × PBS), 184 µl of PBST and 16 µl of BlaR-CTD were added into each well in the first row to reach the final concentration of 20 µg/ml of BlaR-CTD. The plate was covered with paraffin and incubated for 1 h at 37 °C, shaking (~ 150 rpm). After washing with PBST, anti-His tag-HRP conjugated with horseradish peroxidase was diluted 1500-fold in washing buffer and 100 µl/well of the diluted anti-His tag-HRP conjugated was added to all wells. The plate was incubated for 1 h at 37 °C, shaking (~ 150 rpm) followed by washing the plate with PBST. Finally, 100 µl/well of TMB substrate was added to all wells and incubated for 15–30 min at room temperature until the dark blue color was appeared. Immediately after, 100 µl/well of stop solution 2M H_2_SO_4_ was added to stop the reaction and changed the color from dark blue to yellow. The absorbance at 450 nm was performed to detect the binding of BLaR-CTD to the penicillin molecules.

Taking into account that Tetracycline and Kanamycin antibiotics were applied to check the cross reactivity of produced BLaR-CTD with other antibiotics. Furthermore, another recombinant antibody i.e. anti-estrogen receptor antibody were applied as a negative control for the produced BLaR-CTD.

### Formulation of purified recombinant protein

#### Factor optimization via RSM

Seven factors examined in the screening experiment included the concentration of various pH, presence of phosphate and Tris buffers, and the concentration of salts and metal ions (Table 2). The cell density of induction (OD) was also recorded for each factor. Based on PBD results (the PDB file of BlaR-CTD named 1NRF_1 is available at RCBS (https://www.rcbs.com/)),three of the most significant factors (Sucrose, Glycerol and pH) were selected for further optimization by Central Composite Design (CCD) in Design of Experiment (DOE) of Minitab software V17 resulted in twenty- one experimental runs, including three central points. The analyses of experimental data were carried out statistically by regression method (formula 1).

**Table 2:** Seven factors examined and their concentration

N	Excipient	concentration
1	Sucrose	100 mM
		200 mM
		300 mM
		400 mM
2	Glycerol	10%
		15%
		20%
3	Tween-20	0.10%
		0.25%
		0.50%
		1%
4	Triton	0.10%
		0.25%
		0.50%
		1%
5	Borate	5 mM
		10 mM
		15 mM
		20 mM
6	Tris	5 mM
		10 mM
		15 mM
		20 mM
7	PBS	5 mM
		10 mM
		15 mM
		20 mM

1 $$\mathrm{Y }=\upbeta 0 + \sum \mathrm{\beta i} \mathrm{xi }+ \sum \mathrm{\beta iixii} 2 + \sum \mathrm{\beta ijxi xj} +\upvarepsilon$$

Where Y is the predicted OD, β0 is a constant coefficient, βi the linear coefficient, βii the quadratic coefficient and βij the cross-product coefficient. Xi and Xj are input independent variable levels, while ε is the residual error.

To get a response, 100 µl from the stock solution was added to each well of an UV transparent 96-well plate to reach the final concentration of 6 µg/ml BlaR-CTD. The plate was covered with paraffin and incubated for 2h and 6h at 37 °C. The absorbance at 260 nm, 280 nm and 350 nm was measured to check the potential of each excipient in maintenance the stability of the BlaR-CTD from aggregation. After performing experiments, the response changes at time 0 (T0), 2 (T1) and 6 h (T2) were analyzed statistically separately at 350 and 260/280 nm. Model validation parameters and variable significance values were reported in ANOVA (Analysis of variance) and fit statistic tables. Significant variables (*p*-values < 0.05) were selected based on the ANOVA table and main effects plot of response means.

## Result

### Construct Preparation and Transformation evaluation

**Fig. 1:**
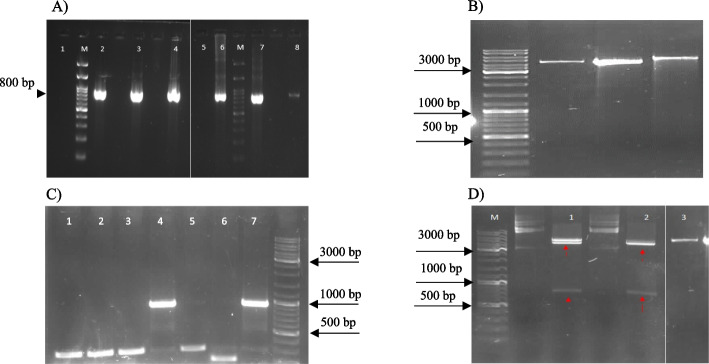
**A** Agarose gel analysis of BlaR-CTD PCR product. Lane 1&5, pcr negative control; M, 500bp DNA ladder; Lane 2, 3, 4, 6, 7, and 8 show PCR product (~ 800 bp) in different concentration. Lane 8 was used for cloning. **B** Agarose gel analysis of pColdII vector PCR with specific primers before insertion. M, 500 bp DNA ladder; Lane 1, pColdI NdeI/HindIII PCR product; lane 2, pColdIV NdeI/HindIII PCR product; and lane 3, pET21a NdeI/HindIII PCR product. There is not any fragment with ~ 800bp length in PCR product. **C** Agarose gel analysis of Colony-PCR with pCold primers. Lane 4&7 are positive colonies for tranformation and other lane are negative colonies. Arrow indicates insert fragment. M, 500 bp DNA ladder. **D** Agarose gel analysis for evaluation of cloning. M, 500 bp marker; lane 1 & 2, double digested pColdII/BlaR-CTD construct, and lane 3, negative control (pColdII vector without insert fragment). Arrow indicates the insert fragment after double digestion

The length of BlaR-CTD PCR product with specific primers (F-NdeI-BLA and R-HindIII-BLA) analyzed by agarose gel electrophoresis, and the optimum concentration for cloning was cleaned up (Fig. 1A ~ 800 bp).

Before insertion step, the pCold II vector was checked with specific primers (Fig. 1B). Then, the pCold II vector and PCR product were digested with NdeI/HindIII enzymes and ligated by T4 ligase. The pCold II/ BlaR-CTD construct was transformed into Bl21 gold (DE3) competent cells. To confirm transformation, colony-PCR with pCold primers was done (Fig. 1C). After positive colonies culture, plasmid extraction and double digestion were performed for cloning validation (Fig. 1D).

### Recombinant BlaR-CTD expression and purification

**Fig. 2:**
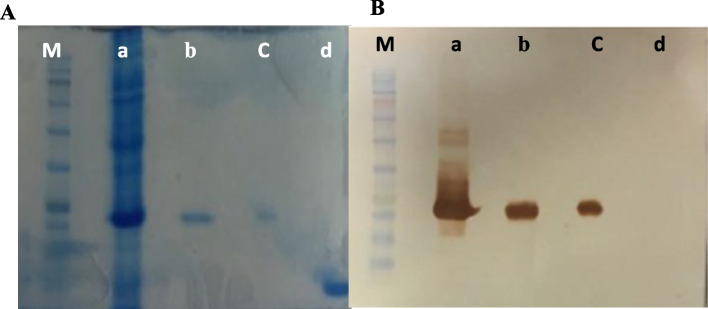
Bacterial extract and purified protein verify by SDS-PAGE (**A**) and western blot (**B**). **a**. Bacteria extract, **b**. Purified receptor, **c**. Purified receptor after changing the buffer, **d**. Negative control, (m). Protein ladder

Three widely considered factors that are known to affect the yield of cytoplasmic soluble protein expression are inducer concentration, temperature, and post-induction incubation duration [11]. The protein expression yield was about 305 µg/ml. Bacterial extract and purified protein were subjected to western blot to confirm the expression and purification. Additionally, after changing the buffer, the observed band was still in the range of 10 to 15 kD (Fig. 2).

### Recombinant BlaR-CTD activity evaluation

To evaluate the activity of the produced recombinant BlaR-CTD i.e. its affinity to bind to the penicillin, ELISA was carried. The absorbance at 450 nm was recorded in triplicate with the average of 2.796 confirmed the high affinity of recombinant BlaR-CTD to the penicillin molecules.

Neither any cross reactivity was observed with other antibiotics like tetracycline and kanamycin, nor any binding of another recombinant antibody i.e. anti-estrogen receptor antibody to the penicillin molecules, which depicted the specificity of the produced recombinant BlaR-CTD to penicillin molecules. The observed absorbance of the blanks was similar to the samples with anti-estrogen receptor antibody (as a negative control for recombinant BlaR-CTD) which was on average about 0.1.

### Recombinant BlaR-CTD stability evaluation

**Fig. 3:**
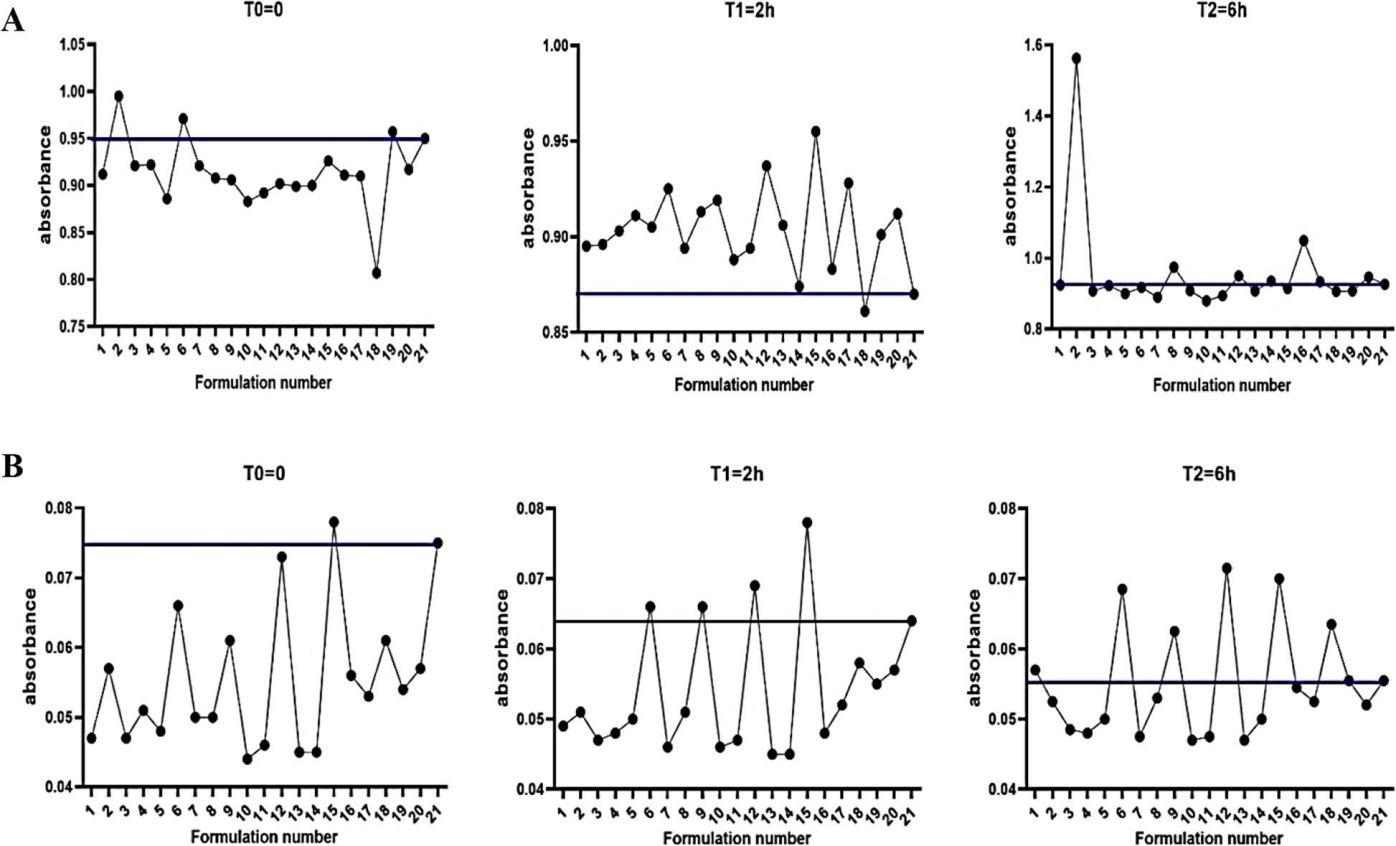
The stability of BlaR-CTD in the presence of 7 different factors analyzed by comparing the absorbance at 260/280 nm (**A**) and 350 nm (**B**)

Based on Fig. 3, the OD related to the 7 factors (Sucrose, Glycerol, Tween-20, Triton, Borate, Tris, and PBS) at ratio 280/260 and 350 nm was measured. The stability of recombinant BlaR-CTD in the presence of 7 different factors with 4 concentrations analyzed in three different wavelengths (280/260 and 350 nm) and pH range 7–9. Ratio of 260/280 nm of BlaR-CTD in T0: 2, 6 and 19, in T1: except 18 and in T2: 2, 8, 16 and 20 other formulation relativity stable to 21. Therefore, 2 and 8 are suitable candidates for stable formulation (Fig. 3A). Regarding to the absorbance at 350 nm, recombinant BlaR-CTD has been relatively stable in T0 except 15, in T1 except 6, 9, 12, 15 and in T2 except 1, 6, 9, 12, 15, 18 in comparison to other formulations (Fig. 3B).

### DOE and CCD

**Fig. 4:**
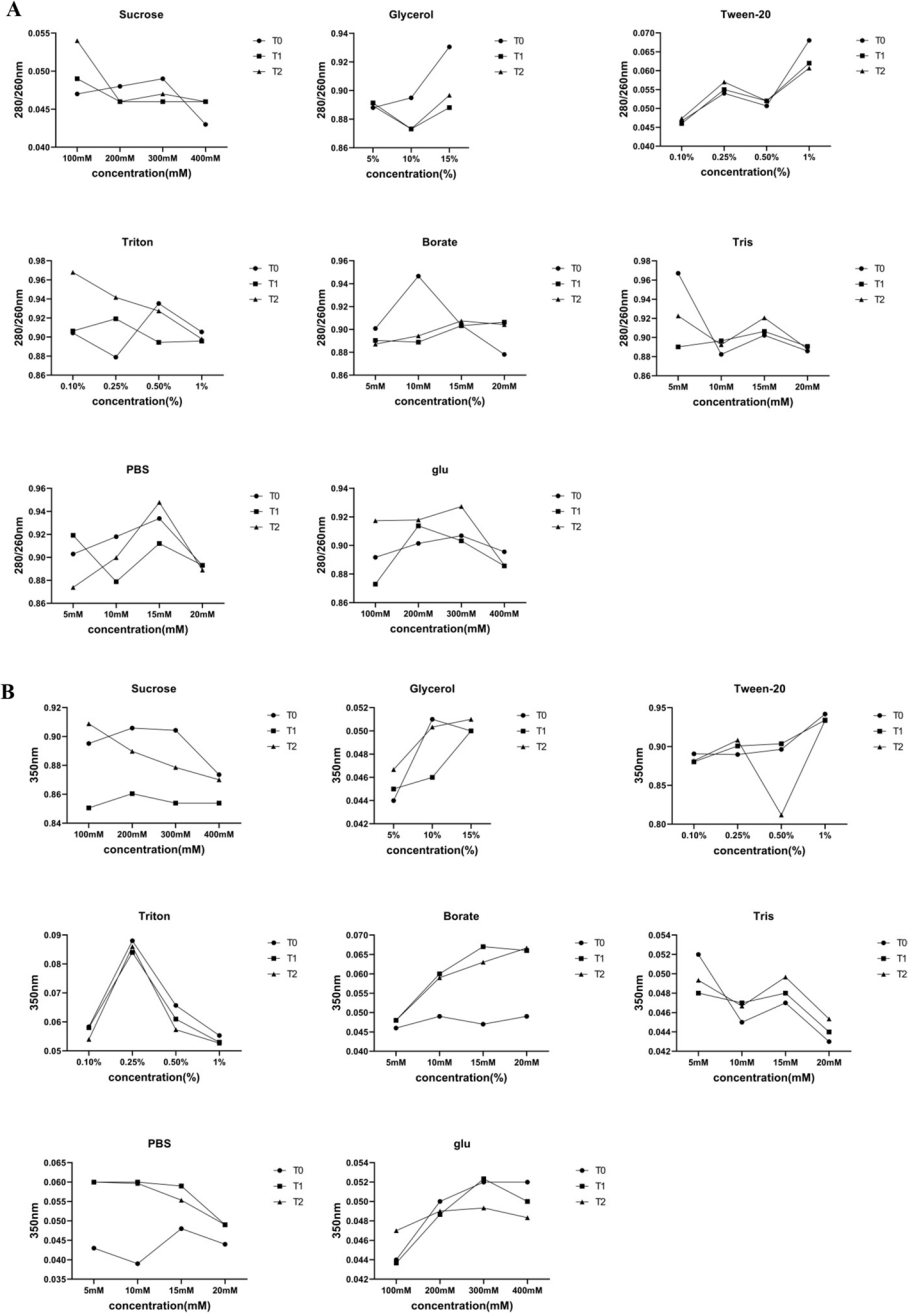
The OD related to the solutions at ratio 280/260 nm (**A**) and 350 nm (**B**) incubated at 37 °C for 0 (T0), 2 (T1) and 6 (T2) hours

The OD related to the solutions include Sucrose, Glycerol, Tween-20, Triton, Borate, Tris and PBS at ratio 280/260 and 350 nm at time 0, 2 and 6 h were recorded. According to DOE, the objective function (response/dependent variable) was wavelengths and the factors (independent variables) were pH, sucrose and glycerol with levels of 7–9, 100–300 mM and 5–20%, respectively. The OD recorded at ratio 280/260 and 350 nm for 20 samples at time 0, 2 and 6 h. The recorded OD are closer to zero and 1 at wavelengths of 350 and ratio 280/260, respectively (Table 3, Fig. 4).

**Table 3:** UV Detection at 350 nm and the ratio of 280/260 nm of control and BlaR-CTD formulated with different excipients

StdOrder	RunOrder	PtType	Blocks	PH	Sucrose (mM)	Glycerol (%)	350 nm			280/260 nm		
							T0	T1	T2	T0	T1	T2
12	1	-1	1	9	100	20	0.047	0.049	0.057	0.912	0.895	0.924
10	2	-1	1	9	300	20	0.057	0.051	0.0525	0.995	0.896	1.012
5	3	1	1	7	100	10	0.047	0.047	0.0485	0.921	0.903	0.907
1	4	1	1	7	300	10	0.051	0.048	0.048	0.922	0.911	0.922
20	5	0	1	7	300	20	0.048	0.050	0.05	0.886	0.905	0.900
13	6	-1	1	9	200	15	0.066	0.066	0.0685	0.971	0.925	0.917
16	7	0	1	6	200	15	0.050	0.046	0.0475	0.921	0.894	0.889
6	8	1	1	9	300	10	0.050	0.051	0.053	0.908	0.913	0.974
14	9	-1	1	8	200	15	0.061	0.066	0.0625	0.906	0.919	0.908
9	10	-1	1	7	100	20	0.044	0.046	0.047	0.883	0.888	0.879
4	11	1	1	8	200	15	0.046	0.047	0.0475	0.892	0.894	0.894
2	12	1	1	8	200	15	0.073	0.069	0.0715	0.902	0.937	0.949
11	13	-1	1	8	200	20	0.045	0.045	0.047	0.899	0.906	0.907
15	14	0	1	8	200	15	0.045	0.045	0.05	0.900	0.874	0.935
3	15	1	1	8	100	15	0.078	0.078	0.07	0.926	0.955	0.914
18	16	0	1	8	200	15	0.056	0.048	0.0545	0.911	0.883	1.049
17	17	0	1	8	300	15	0.053	0.052	0.0525	0.910	0.928	0.933
8	18	1	1	8	200	20	0.061	0.058	0.0635	0.807	0.861	0.906
19	19	0	1	9	100	10	0.054	0.055	0.0555	0.957	0.901	0.907
7	20	1	1	8	300	10	0.057	0.057	0.052	0.917	0.912	0.946
21	21	1	1	7	0	0	0.075	0.064	0.0555	0.950	0.870	0.926

In addition, the predicted levels of OD at ratio 280/260 and 350 nm (6 h) in formulas 2 and 3 were calculated based on the equation obtained by regression method:

2 $$\begin{array}{c}280/260\left(6{\text{h}}\right) = -4.46 +1.28\mathrm{ pH }+0.00037\mathrm{ Sucrose }+0.049\mathrm{ Glycerol }-0.079 {{\text{pH}}}^{*}{{\text{pH}}}^{*}\\ - 0.000002 {{\text{Sucrose}}}^{*}\mathrm{ Sucrose}-0.00111 {{\text{Glycerol}}}^{*}{\text{Glycerol}} -0.000030 {{\text{pH}}}^{*}{\text{Sucrose}}\\ - 0.0028 {pH}^{*}Glycerol +0.000035 {{\text{Sucrose}}}^{*}{\text{Glycerol}}\end{array}$$

3 $$\begin{array}{c}350 \left(6{\text{h}}\right) =0.139-0.0122 \mathrm{pH }+0.000651\mathrm{ Sucrose }-0.01638\mathrm{ Glycerol }+0.00125 {{\text{pH}}}^{*} {\text{pH}}\\ - 0.000000 {{\text{Sucrose}}}^{*}\mathrm{Sucrose }+0.000325 {{\text{Glycerol}}}^{*}\mathrm{Glycerol }-0.000076 {{\text{pH}}}^{*}{\text{Sucrose}}\\ + 0.000817 {{\text{pH}}}^{*}\mathrm{Glycerol }-0.000000 {{\text{Sucrose}}}^{*}{\text{Glycerol}}\end{array}$$

According to Table 4, the analysis of variance (ANOVA) based on the DOE and CCD design for 350 and 280/260 wavelengths, respectively. It was shown that sucrose at 350 nm (*P*-value = 0.006) and pH at ratio 280/260 nm (*P*-value = 0.016) have a significant effect on the wavelength. In addition, the quadratic effect of sucrose*sucrose (*P*-value = 0.023) at 350 nm and pH*pH (*P*-value = 0.033) at 260/280 nm were also significant.

**Table 4:** Analysis of variance based on CCD

Source	350 nm					260/280 nm				
	DF	Adj SS	Adj MS	F-Value	*P*-Value	DF	Adj SS	Adj MS	F-Value	*P*-Value
Model	9	0.000078	0.000009	3.06	0.048	9	0.187504	0.020834	1.96	0.154
Linear	3	0.000038	0.000013	4.44	0.031	3	0.089839	0.029946	2.82	0.093
pH	1	0.000003	0.000003	1.08	0.322	1	0.089545	0.089545	8.43	0.016*
Sucrose	1	0.000034	0.000034	11.89	0.006*	1	0.000035	0.000035	0.00	0.956
Glycerol	1	0.000001	0.000001	0.35	0.567	1	0.000260	0.000260	0.02	0.879
Square	3	0.000024	0.000008	2.77	0.097	3	0.095751	0.031917	3.00	0.081
pH*pH	1	0.000002	0.000002	0.80	0.392	1	0.065304	0.065304	6.15	0.033*
Sucrose*Sucrose	1	0.000020	0.000020	7.23	0.023*	1	0.013046	0.013046	1.23	0.294
Glycerol*Glycerol	1	0.000001	0.000001	0.25	0.628	1	0.008111	0.008111	0.76	0.403
2-Way Interaction	3	0.000017	0.000006	1.97	0.183	3	0.001913	0.000638	0.06	0.980
pH*Sucrose	1	0.000010	0.000010	3.57	0.088	1	0.000190	0.000190	0.02	0.896
pH*Glycerol	1	0.000006	0.000006	2.16	0.172	1	0.000903	0.000903	0.09	0.777
Sucrose*Glycerol	1	0.000000	0.000000	0.18	0.683	1	0.000820	0.000820	0.08	0.787
Error	10	0.000028				10	0.106235	0.010624		
Lack-of-Fit	5	0.000017	0.000003			5	0.095928	0.019186	9.31	0.014
Pure Error	5	0.000012	0.000002	1.40	0.362	5	0.010307	0.002061		
Total	19	0.000106				19	0.293739			

**Fig. 5:**
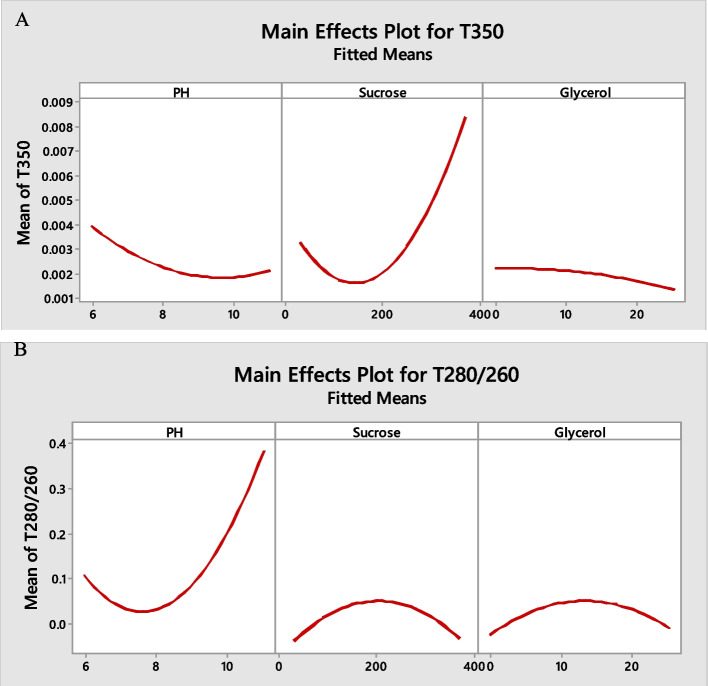
Main effect plots for 350 nm (**A**) and ratio 280/260 nm (**B**)

Figure 5A shows the main effect plots where at 350 nm the slope of the graph of pH and glycerol was almost downward and the graph of sucrose was non-linear. This means that with the increase of pH and glycerol, the recorded wavelength decreased, but the lowest wavelength was recorded for sucrose at a concentration of less than 200 mM. Also, at ratio 280/260, with increasing pH, the highest wavelength was recorded. Regarding sucrose and glycerol, the highest wavelength were at the concentration of 200 mM and 15%, respectively (Fig. 5B).

**Fig. 6:**
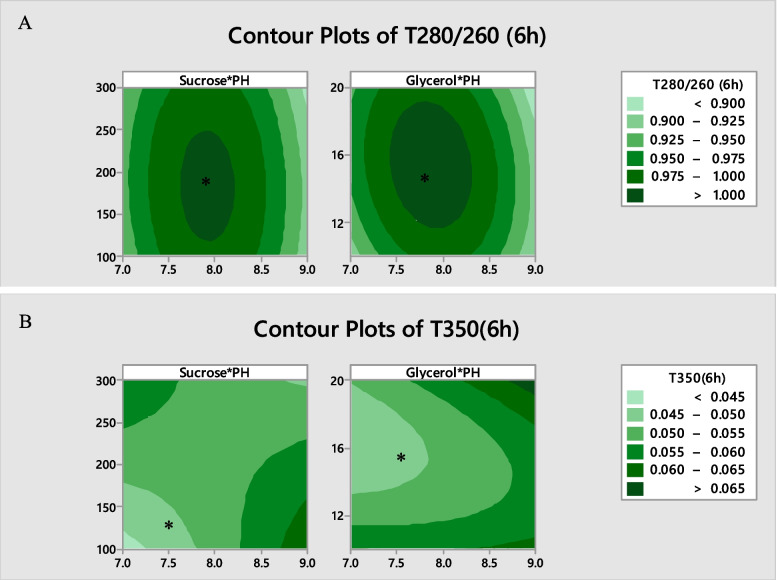
Contour plot of ratio 280/260 nm (**A**) and 350 nm (**B**) for OD at optimal level of sucrose*pH and glycerol*pH (the stars indicate optimal formulations)

Figures 6A and B represent the contour plots for the two wavelengths at the levels of pH and sucrose besides pH and glycerol. The predicted optimal constituents are indicated by stars in these figures. In such a way these areas are close to 1 at 280/260 wavelength and close to zero at 350 wavelengths. Therefore, the optimal wavelength was obtained at ratio 280/260 and 350 wavelengths, based on the maximum and minimum value of the recorded wavelength, respectively.

**Fig. 7:**
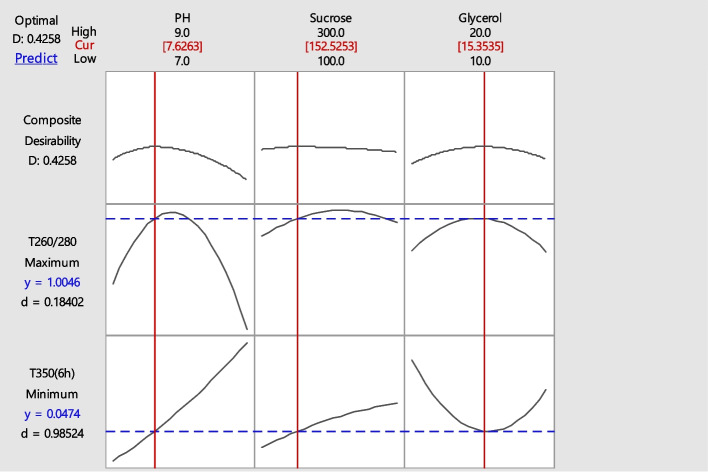
Optimization plot for the highest activity of recombinant BlaR-CTD

As shown in Fig. 7, the maximum and minimum wavelengths was determined 1.0046 at ratio 280/260 nm and 0.0474 at 350 nm, respectively. Based on these results, the optimal levels of pH, sucrose and glycerol were predicted as 7.6262, 152.5253 and 15.3535 respectively.

## Discussion

Attaining an optimum formulation for a recombinant protein could be influenced by different factors [23, 24]. Since one of the biggest concerns of the biopharmaceutical manufacturing is the cost-effectiveness and repeatability of the recombinant protein production process, chemically defined-minimal media as excellent solutions can be used to achieve these two goals [25, 26]. Several studies have attempted to achieve maximal production under optimal conditions. Despite the constructive benefits of utilizing *E. coli*, high-level synthesis of soluble recombinant proteins can be challenging. Aggregated proteins, known as inclusion bodies, are generally misfolded and physiologically inactive [27]. In many cases, solubilization and refolding of insoluble proteins are time-consuming and labor-intensive procedures that add to the complexity of downstream processing [28, 29]. Several methods have been developed to address these challenges to increase the solubility of recombinant proteins. For instance, an efficient and practical method is to modify culture conditions [30]. According to the accumulated evidence to accelerate the expression of target genes and develop solubility in *E. coli*, remarkable methods have been developed. Four substantial factors highly affect the production of soluble protein: timing of induction, inducer concentration, temperature, and duration of induction. Nonetheless, efforts to identify optimal buffer conditions frequently rely on re-purification or functional assays, a time- and protein-intensive trial-and-error strategy. An optimal time window exists for the production of proteins. The correlation between the growth rate and the specific protein production rate for the induced batch and fed-batch cultures suggests that a sluggish growth rate under induced conditions produces little or no product. In addition, the duration of expression was limited in batch cultures. This provides information on protein aggregation in the cell and protein quality [31]. Induction is typically conducted during the early mid-log phase, although there are reports of induction occurring during the late-log phase or even during the stationary phase [32].

The recombinant nature of protein drugs allows intervening at upstream stages through protein engineering to produce analogue protein versions with higher stability and enhanced therapeutic values. This highlights the importance of protein engineering and structural analysis in improving the stability of recombinant proteins such as BlaR-CTD. In a study performed by Jianan et al., the stability of BlaR-CTD and its mutant proteins has been compared, revealing insights into their structural and functional properties. The wild-type BlaR-CTD was found to exhibit poor stability, while certain mutant proteins showed enhanced stability [7]. However, changing the recombinant protein structure using point mutations is not favorable in industry as this mutant/ modified protein is not anymore biosimilar therapeutic.

Another approach is to alter process conditions to maximize the wild-type construct stability based on a specific protein stability profile (PSP). A systematic analysis of pH, buffer or salt identity and concentration, biological metals, surfactants, and common excipients in terms of an effect on protein stability rapidly identifies conditions that might be used (or avoided) during protein production [33].

Protein-based therapies are complicated products with obstacles in obtaining stability from manufacturing to patient administration. To obtain stability of recombinant proteins, various strategies can be employed. Several solutions have been investigated, ranging from formulation modification to the invention of delivery systems, in an attempt to solve biopharmaceutical stability limits and safety issues as well as increase product quality and patient compliance.

Additionally, protein aggregation during purification can lead to remarkable solubility issues. Furthermore, proteins are susceptible to solution concentration, whereas recombinant protein aggregates can be solubilized during purification using a variety of buffer conditions [34]. Thus, an effective screening buffer should be considered at various concentrations and under optimal conditions to obtain specific results.

Excipients play a crucial role in stabilizing protein formulations and minimizing potential aggregation. They are additives that help stabilize the protein's structure and reduce unwanted aggregation cascade. Excipients can range from simple buffers to more complex components such as amino acids, sugars, surfactants, and antioxidants. Their mechanisms of action include strengthening protein-stabilizing forces, destabilizing the denatured state, and direct binding to the protein. For instance, surfactants, or surface-active agents, play a major role in preventing protein aggregation [35, 36]. During the manufacturing, storage, and delivery processes, therapeutic proteins encounter various surfaces and materials, such as metals, glasses, oils, and polymers. The inherent surface-active nature of proteins can cause them to interact with surfaces, leading to possible denaturation and subsequent aggregation. Excipients help mitigate these interactions and prevent aggregation, thereby ensuring the stability and quality of the protein formulation [35].

It is important to note that the effects of excipients on protein aggregation during agitation can depend on the balance between the excipient's effects on the conformational stability of the native protein in the bulk solution and the extent of protein gelation at air–water interfaces. Therefore, the selection and use of excipients should be carefully considered to achieve the desired stabilization and minimize aggregation [37]. Various excipients can be used to improve the stability of recombinant proteins. To improve the thermal stability of BlaR-CTD, several excipients were analyzed in this research. On this basis, we used US7745193B2 to design our construction. We produced BlaR-CTD in-house to have an access to bulk recombinant BlaR-CTD for investigating the effect of several factors on the stability of this recombinant protein. Therefore, one of the most innovative parts of this study is to assess the optimum concentrations of several excipients to achieve high thermal stability of recombinant BlaR-CTD.

The conventional method to optimize the formulation is to change one parameter at a time while maintain the other constants. However, because of the high number of required experiments due to multiplex parameters, this approach is not feasible. In addition to the inconvenient nature of this approach, if there is an interaction between different variables, it can cause misinterpretation of the results [17, 38]. RSM is a popularly employed alternative procedure to overcome the aforesaid obstacles. It is a mathematic and statistical utilized to design optimization experiments, to create models, and to evaluate the interplays within different bioprocess parameters; whereas reducing the number of possible experiments [39].

To optimize the formulation of recombinant BlaR-CTD, we designed an experimental RSM/CCD methodology that included seven different factors (Sucrose, Glycerol, Tween-20, Triton, Borate, Tris, and PBS) with four concentrations measured at three different wavelengths (280/260 and 350 nm) and a pH range of 7–9. The point prediction tool of the software was utilized to calculate the optimum value of concentration sucrose (152.5 mM), glycerol (15.3%), and pH (7.6). Eventually, a quadratic model (Eq. 1) was proposed by DOE Minitab software based on the experimental results. The developed model showed that among the three mentioned variables, OD recorded for sucrose and pH had a significant effect in 350 nm and 280/260 nm, respectively.

However, it is suggested that not only further excipients but also other parameters influencing protein stability should be investigated to obtain a higher stability of recombinant BlaR-CTD protein. Therefore, in our upcoming experimental studies, we will assess further on the stability of this recombinant protein.

## Conclusion

The results of this study showed that the maximal thermal stability of recombinant BlaR-CTD protein expressed in *E. coli* were obtained with glycerol 15.35%, sucrose 152.52 mM, and pH 7.62. Particularly, Sucrose in 350 nm and pH in the ratio of 280/260 nm using RSM/CCD methodology. Among seven different factors including Sucrose, Glycerol, Tween-20, Triton, Borate, Tris, and PBS besides a pH range of 7–9, sucrose and pH depicted the greatest effect on the stability of recombinant BlaR-CTD protein.

## Data Availability

Yes.
